# Newborn Screening for Methylmalonic Acidemia in a Chinese Population: Molecular Genetic Confirmation and Genotype Phenotype Correlations

**DOI:** 10.3389/fgene.2018.00726

**Published:** 2019-01-23

**Authors:** Wei Zhou, Huizhong Li, Chuanxia Wang, Xiuli Wang, Maosheng Gu

**Affiliations:** Xuzhou Maternity and Child Health Care Hospital, Xuzhou, China

**Keywords:** methylmalonic acidemia, newborn screening, *MMACHC*, *MUT*, MS/MS

## Abstract

**Background:** Methylmalonic acidemia (MMA) incidence was evaluated based on newborn screening in Xuzhou from November 2015 to December 2017, and the clinical, biochemical and molecular characteristics of patients with MMA harboring *MMACHC* and *MUT* mutations were summarized.

**Methods:** During the study, 236,368 newborns were screened for MMA by tandem mass spectrometry (MS/MS) in the Maternity and Child Health Care Hospital of Xuzhou. C3, C3/C2 and methionine, and tHcy if necessary, were measured during the first screening. Blood samples from the infants and/or their family members were used for DNA analysis. The entire coding regions of the *MMACHC* and *MUT* genes associated with MMA were sequenced by DNA MassARRAY and next-generation sequencing (NGS).

**Results:** Eleven patients with *MMACHC* mutations and three with *MUT* mutations were identified among the 236,368 screened newborns; the estimated total incidence of MMA was 1:16,883. Among the MMA patients, two died of infection-triggered metabolic crisis approximately 3 months after birth. All the patients identified had two mutant alleles except for one individual with early-onset disease. The most common *MMACHC* mutation was c.609G > A. The laboratory levels of C3 and C3/C2 were elevated in MMA individuals compared to other infants. Importantly, we demonstrate that accelerated C2 degradation is related to air temperature and humidity.

**Conclusion:** Our study reports the clinical characteristics of MMA and diagnosis through MS/MS and NGS. There was a higher incidence of MMA with homocysteinemia than of isolated MMA in Xuzhou. Insight from this study may help explain the high false-positive rate of MMA in summer.

## Introduction

**FIGURE 1:**
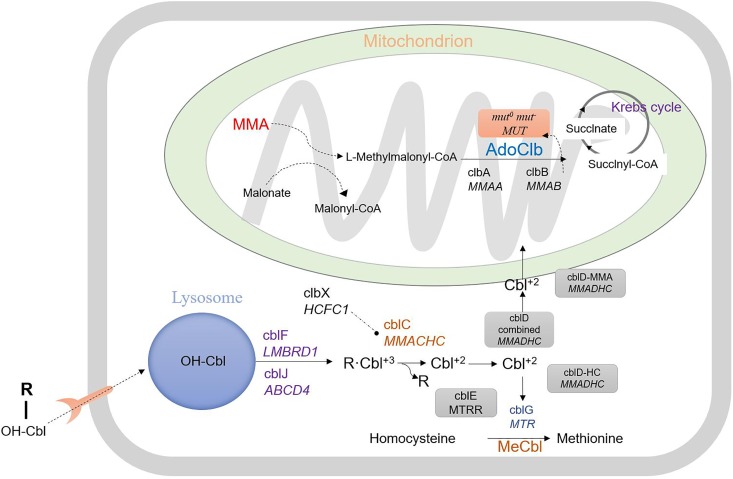
Intracellular vitamin B12 (or cobalamin) metabolism.

Methylmalonic acidemia (MMA) comprises a series of autosomal recessive inherited disorders of organic acid metabolism. The primary defect lies in methymalonyl-CoA mutase (MCM) or its cofactor, adenosylcobalamin (AdoCbl) (Han L. et al., 2015). MMA causes various clinical symptoms including recurrent vomiting, metabolic acidosis, and even developmental delay (Radmanesh et al., 2008). The cellular pathway in MMA by which cobalamin (OH-Cbl) is processed to AdoCbl and methylcobalamin (MeCbl) is depicted in Figure 1. The cobalamin-processing enzymes have been indicated to be causative factors (Obeid et al., 2015; Richard et al., 2018). MMA can be classified into two common types: isolated MMA and combined MMA and homocysteinemia, which are caused by deficiency in the *MUT* or *MMACHC* gene, respectively. In addition to the two above-mentioned MMA types, there are five other less common subtypes, including cblD, cblF, cblJ, and cblX deficiencies (Yu et al., 2013; Hu et al., 2018). Moreover, some other mutant genes associated with isolated MMA have also been verified including *MMUT, MMAA, MMAB, MCEE*, and *MMADHC* (Hörster et al., 2007; Ktena et al., 2015; Peng et al., 2018). The most common biochemical hallmark of these defects is the accumulation of methylmalonic acid and homocysteine in the blood and urine due to the dysfunction in MeCbl and AdoCbl (Carrillo-Carrasco et al., 2012; Huemer et al., 2014).

Isolated MMA is characterized by the abnormal accumulation of methylmalonic acid in metabolites without hyperhomocysteinemia. The *MUT* gene lies within chromosome 6p12.3 and has 13 exons that encode 750 amino acids (Worgan et al., 2006). Approximately 250 mutations have been identified to date in the *MUT* gene in different populations. The clinical manifestations and biochemical parameters of isolated MMA patients commonly present during the first weeks and months after birth as poor feeding, recurrent vomiting, and severe metabolic acidosis.

Combined MMA and hyperhomocysteinemia, cblC type, is the most common inherited disorder of cobalamin metabolism, and the related gene, *MMACHC*, is located at 1p34.1 (Weisfeld-Adams et al., 2013; Pastore et al., 2014). CblC disease patients usually exhibit systemic and neurological abnormalities in the neonatal period. Existing research recognizes the critical role of timely disease treatment.

Data obtained from various countries worldwide suggest that the estimated incidence of MMA ranges from 1:48,000 to 1: 250,000 (Weisfeld-Adams et al., 2013). Wang et al. (2010) reported the incidence of cblC disorder by newborn screening to be in the high range of 1: 3,920 for an ethnically diverse population in Shandong, China, contrasting with a previous estimate of 1:28,000 births by clinical ascertainment in Shanghai (Han et al., 2016). The introduction of an expanded screening program for newborns is recommended to identify those infants who are considered at risk for MMA. Now that an MMA diagnosis can be confirmed by DNA testing and gas chromatography/mass spectrometry (GC/MS) based urine analysis, it is possible to improve the outcome of newborn screening for MMA. With regards to the work conducted in our center, newborn screening for MMA began in November 2015 in Xuzhou, Jiangsu Province, China. We documented the clinical features and spectrum of gene mutations in 15 *MUT* and *MMACHC* patients and identified 14 mutations, including 5 *MUT* and 9 *MMACHA* mutations. We aimed to estimate the incidence of MMA during newborn screenings and to summarize the clinical features, molecular characteristics, treatment, and follow-ups of infants with MMA who received early treatment.

## Patients and Methods

### Study Population and MS/MS Newborn Screening

From November 2015 to December 2017, 236,368 newborns were screened for PCD at the Genetic Medicine Center of the Maternity and Child Health Care Hospital in Xuzhou. Acylcarnitine concentrations were quantified by tandem mass spectrometry (MS/MS), with a quantify spot of 2 mg/dL (134 μmol/L) for whole blood. The reference levels of propionylcarnitine (C3), C3/acetylcarnitine (C2), and methionine were measured. 52 among the 236,368 newborns were included in the recalling screening and 14 individuals were involved with confirmatory testing. Informed and written consent was obtained from the guardians of all the patients before clinical testing.

### DNA Sequencing and Diagnosis

Blood samples from infants with an abnormal newborn screening and/or their family members were sent to Bioscan Genomics, Hangzhou. Subsequently, DNA was extracted from peripheral blood leukocytes using the OMEGA Genomic DNA Extraction Kit (OMEGA Biotech, United States). The targeted DNA sequence was mapped to and analyzed against a genetic diagnosis panel of inherited metabolic diseases associated with the *MMACHC* and *MUT* genes, including *MMAA*, *MMAB*, *MMACHC*, *MUT*, *PCCA*, and *PCCB*.

To further confirm the diagnosis, the levels of organic acids in the urine were measured with GC/MS. Moreover, the concentration of total plasma homocysteine (tHcy) was determined (Weisfeld-Adams et al., 2010). All tests were performed after informed consent was obtained from the guardians of all the patients.

### Treatment and Follow-Up

The patients with clbC type disease were given hydroxocobalamin (OHCbl) (1–2 mg, two-three times weekly, intramuscular injection), levocarnitine (100 mg/kg/day, oral administration), betaine (250 mg/day, oral administration), and folinic acid (5–15 mg/day, oral administration). Levocarnitine was administered in conjunction with restricted protein intake to most isolated MMA individuals in accordance with disease progression.

Outpatient clinical follow-up occurred once per month during the initial treatment, followed by once every 3 months or longer once stable conditions were achieved. Changes in MMA, C3, tHcy and methionine levels and mental development were monitored during treatment. Infants with MMA were followed up for 1.8 years after birth to the present.

### Statistical Analysis

In the statistical analysis, *t*-tests were used to analyze the effects of changes in the experimental condition. A *P*-value < 0.05 was considered significant.

## Results

### Clinical and Biochemical Description

Fourteen patients were identified by screening 236,368 newborns, yielding an estimated MMA incidence of 1:16,883 live births, while the incidence of *MMACHC* cblC and isolated *MUT* MMA was 1:19,697 vs. 1:78,789, respectively. All the patients presented with elevated C3 levels (>5 μmol/L), an increased C3/C2 ratio (>0.18), and low or normal methionine levels in dried blood spots (normal range 5–40 μmol/L) (Tables 1, 3). It is worth mentioning that the reference interval was in accordance with the percentiles of 200,000 health screening samples in Xuzhou. Subsequently, the normal range of methionine was confirmed. Indeed, there exists a mild difference of the cut-off value worldwide (Weisfeld-Adams et al., 2010). In addition, the patients with clbC disease exhibited increased methylmalonic acid levels (>5 mg/gCr) in the urine and elevated total plasma homocysteine (>13.56 μmol/L) (Table 1). 11 of the 14 individuals were diagnosed with combined MMA and hyperhomocysteinemia, and the diagnoses were confirmed by *MMACHC* gene sequencing. However, data about tHcy levels are limited and insufficient partly due to patient referral. Further work of data collection is being performed to confirm this finding. Another three patients, shown in Table 3, were diagnosed with isolated MMA at ages varying from 3 days to 4 weeks with DNA sequencing reports, while they were all received immediate intervention after newborn screening. As can be seen from Table 3, high levels of blood C3 and urinary MMA were detected. Taken together, the data showed that all three of these patients survived with developmental delay after receiving timely treatment after diagnosis.

**Table 1:** Biochemical data and DNA mutations features of eleven patients with *MMACHC*-type diagnosed on newborn screening.

Case	Gender	Age at diagnosis (time after birth)	Birth weight (kg)	C3 on NBS (μmol/L)	C3/C2 ratio on NBS	Met on NBS (μmol/L)	Urine MMA (mg/gCr)	THcy (μmol/L)	*MMACHC* genotype	Variation in protein level
1	F	20 days	3.75	4.04	0.24	7.40	158.74	89.60	c.482G > A/c.658_660delAAG	p.D77Qfs^∗^22/p.K220del
2	M	24 days	3.45	7.70	0.46	11.95	33.48	109.5	c.1A > G/c.658_660delAAG	p.M1V/ p.K220del
3	F	3 weeks	2.50	5.21	1.57	4.71	–	–	c.276+1G > A/c.658_660delAAG	//p.K220del
4	M	20 days	2.95	8.19	0.24	11.05	5.82	102.36	c.609G > A/c.228_231delTGAC	p.W203^∗^/p.D77Qfs^∗^22
5	M	15 days	3.00	6.65	0.91	8.67	12.32	92.32	c.567dupT/c.80A > G	p.I190Yfs^∗^13/p.Q27R
6^∗^	F	10 days	3.30	7.92	0.35	19.95	66.35	–	c.482G > A/-	p.R161Q/-
7	F	4 weeks	1.95	6.57	0.38	9.89	163.84	41.99	c.609G > A/c.567dupT	p.W203^∗^/p.I190Yfs^∗^13
8	M	20 days	3.65	7.21	0.39	17.06	46.73	86.32	c.482G > A/c.609G > A	p.R161Q/ p.W203^∗^
9	F	24 days	2.70	6.42	0.85	6.09	155.09	–	c.609G > A/c.609G > A	p.W203^∗^/ p.W203^∗^
10	M	3 weeks	3.80	8.05	0.43	16.79	21.22	–	c.482G > A/c.515A > G	p.R161Q/ p.K172T
11	M	18 days	2.40	8.8	1.17	4.87	46.93	–	c.567dupT/c.609G > A	p.I190Yfs^∗^13/p.W203^∗^


*F, female; M, male; ^∗^, this case who was identified with only one mutation in MMACHC gene but this patient with typical detected results of blood by MS/MS and urine by GC/MS, so the case might be considered as MMA patient in clinic with some undetected mutations. And we would like to identify the gene mutation in some other methods in the future. Age at diagnosis was identified with DNA reports.*

Six patients started treatment immediately following confirmation of the clbC disease diagnosis. Unfortunately, case 3 died from metabolic crisis triggered by infection approximately 1 month after birth. Cases 9, 10, and 11 were lost to follow-up, most likely due to population mobility. The other patients are alive. Five patients (cases 2, 4, 5, 7, and 8) were treated with intramuscular hydroxocobalamin, levocarnitine, betaine and folinic acid (Table 2). Case 1 was treated similarly, to the above patients but without betaine. Case 12 was given intramuscular hydroxocobalamin alone (Table 2). All the patients who received treatment had a favorable metabolic response, with reduced urine MMA and tHcy and increased methionine (Table 2).

**Table 2:** Therapeutic regime, biochemical data and clinical features in patients with *MMACHC*-type after treatment.

Case number	Therapeutic regime				Clinical development estimation	Metabolic parameters at recent visit			
			
	OHCbl IM	L-carnitine oral (mg/kg/day)	Betaine oral (mg/kg/day)	Folinic acid oral (mg/day)		C3 (μmol/L)	Urine MMA (mg/gCr)	THcy (μmol/L)	Met (μmol/L)
1	1 mg, twice weekly	100	–	7.5	Mild developmental delay	2.10	6.08	15.68	43.4
2	1–2 mg, third weekly	100	250	5	Dead	7.96	39.72	–	58.56
4	1–2 mg, third weekly	100	250	7.5	Severe developmental delay	6.61	29.41	11.66	21.19
5	1–2 mg, third weekly	100	250	7.5	Slight hypotonia, mild developmental delay	7.46	11.12	10.69	38.74
7	1 mg, third weekly	100	250	15	developmental delay	4.94	24.79	52.36	5.65
8	1–2 mg, twice weekly	100	250	7.5	Mild developmental delay	5.58	5.51	10.45	29.25


*PS, developmental delay for young children <4–6 years and intellectual impairment/disability for older children, while given the children are all <2 years old.*

**Table 3:** Clinical characteristics of the *MUT*-type patients.

Case number	Gender	Age at diagnosis	Clinical manifestations	C3 on NBS (μmol/L)	C3/C2 ratioon NBS	Urine MMA (mg/gCr)	Prognosis	*MUT* genotype(Variation in protein level)
P1	M	4 weeks	Feeding difficulty, vomiting	6.77	0.22	40.54	1 y and 3 m, developmental delay	c.441T > A/c.1880A > G (p.D147E/ p.H627R)
P2	M	9 days	Feeding difficulty, hypotonia	8.42	0.71	155.20	10 m, developmental delay	c.1106G > A/c.581C > T (p.R369H/ p.P194L)
P3	F	3 days	No obvious symptoms except vomiting	7.86	1.04	416.00	8 m, ND	c.1106G > A/c.1741C > T (p.R369H/ p.R581^∗^)


*F: female; M: male; y, year; m, month; ND, normal development; ND, no data; Developmental delay for young children <4–6 years and intellectual impairment/disability for older children, while given the children are all <2 years old.*

**FIGURE 2:**
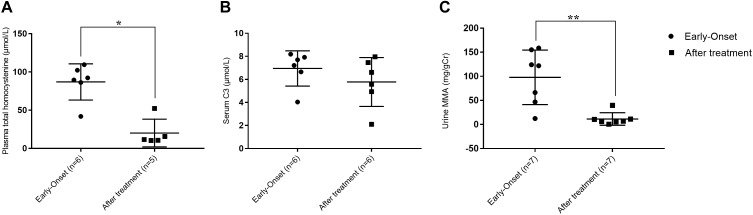
Metabolite measurement of clbC patients. **(A)** Total homocysteine of plasma. **(B)** The level of C3. **(C)** The level of MMA in urine. ^∗^*P* < 0.05, ^∗∗^*P* < 0.01.

Although not all of the biochemical parameters reached the levels present in healthy individuals, laboratory values after treatment were lower than those found in individuals with early-onset clbC. As shown in Figure 2, MMA in urine and total plasma homocysteine dramatically decreased after treatment. C3 in the blood declined but did not reach the normal range.

### MS/MS Biochemical Quantification for MMA

**FIGURE 3:**
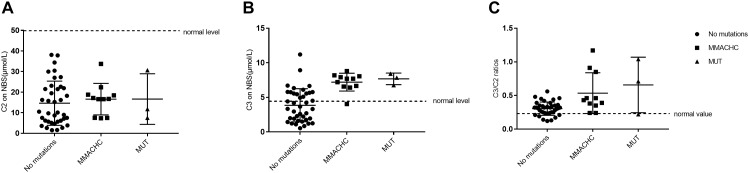
Metabolite measurement in MMA compared to normal, *MMACHC*, and *MUT* patients. **(A)** The level of C2 on newborn screening. **(B)** The level of C3 on newborn screening. **(C)** The C3/C2 ratio on newborn screening.

Of the 236,368 newborns screened by MS/MS in our study, 52 (0.02%) had C3 levels and a C3/C2 ratio exceeding the cutoff values and were reported as screening positive for MMA. According to further diagnostic testing, cases of clbC disease with mutations in the *MMACHC* gene (*n* = 11), isolated MMA with mutations in the *MUT* gene (*n* = 3) and no mutations (*n* = 38) were then reported and treated in our center. Remarkably, to avoid misdiagnosis of other diseases associated with increased C3 in newborn screening, the 38 individuals with no MMA-related mutations underwent DNA sequencing analysis of a genetic diagnosis panel covering 51 diseases and 98 genes. After NGS and data analysis, it was possible to exclude these diseases. Then, these individuals were selected as the control group. However, further data collection is required to determine whether the laboratory results are due to other rare forms of MMA; almost all of the control individuals were followed up for a period after recalling, and the results were normal at the last follow-up. 2-Methylcitric acid (C2) was retrospectively measured in all positive infants (*n* = 52) and ranged from 1.2 to 38.12 μmol/L. Table 4 shows the levels of C2, C3, and C3/C2 in our study patients. As shown in Figure 3, C3 and C3/C2 were primarily elevated in MMA patients, while C2 levels were not different between healthy and MMA newborns. The laboratory levels of C3 and C3/C2 in newborns with early-onset MMA were above normal, as shown in Figure 3, but the distribution in the control group ranged both above and below normal.

**Table 4:** Median and range levels of C2, C3, and C3/C2 obtained from MMA screening.

Confirmed diagnosis	*n*	C2 Mean (Range)(μmol/L)	C3 Mean (Range) (μmol/L)	C3/C2 Mean (Range)
*MMACHC* mutation types	11	17.52 (7.31–33.71)	7.05 (4.04–8.80)	0.64 (0.24–1.57)
*MUT* mutation types	3	16.65 (7.53–30.64)	7.68 (6.77–8.42)	0.66 (0.22–1.04)
No mutations	38	14.25 (1.2–38.12)	3.86 (0.54–11.21)	0.30 (0.12–0.56)


### Accelerated Degradation of MMA Markers

**FIGURE 4:**
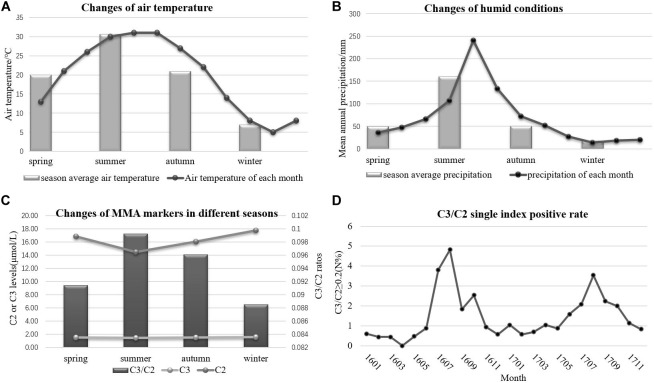
The variation of environmental conditions and MMA markers. **(A)** The changes of air temperature. **(B)** The changes of humidity. **(C)** The changes of MMA markers in different seasons. **(D)** The single index positive rate of MMA.

The accelerated degradation of MMA markers, including C2, C3, and C3/C2, was analyzed while monitoring the effects of heat and humidity on newborn screening tests. Figures 4A,B present the changes in heat and humidity from spring to winter in two recent years in Xuzhou. The air temperature and precipitation were much higher in summer than in other seasons. C2 levels declined sharply during the summer in Xuzhou, but this decline was not observed in C3. However, the C3/C2 ratios shown in Figure 4C were higher in summer than in other seasons, and this marker is crucial for identifying MMA by MS/MS. The thermal stability data for C2 revealed degradation at high air temperature with high humidity. The C3/C2 ratio is considered the single index positive marker for MMA diagnosis, and the data in Figure 4D illustrate its volatility in different months, with higher values in summer, a time of high air temperature and humidity. In the following section, it will be argued that the dried blood spot collection conditions play an important role in newborn screening tests. Several hospital samples showed much higher values than others during the same period due to the high heat and humidity when collecting the blood spots from the infants. After the Cold-Chain Logistics system was implemented in the following year, the single index positive marker C3/C2 sharply declined under the control range (Table 5). Indeed, the *P* value illustrated the importance of changing the collection conditions.

**Table 5:** The changes of ingle index positive rate marker C3/C2 in several hospitals in our study.

Hospital name	Total samples (in 2 years)	2016.08				2017.08				
				
		C3/C2 < 0.2		C3/C2 ≥ 0.2		C3/C2 < 0.2		C3/C2 ≥ 0.2		*P*-value
						
		Samples	*N*%	Samples	*N*%	Samples	*N*%	Samples	*N*%	
Suining People’s Hospital	10874	566	91.14	55	8.86	504	96.18	20	3.82	0.002
Xinyi People’s Hospital	9110	372	88.57	48	11.43	544	98.73	7	1.27	
Pizhou Tiefu Town Health Center	2721	120	72.29	46	27.71	159	96.95	5	3.05	


### Mutation Analysis and Molecular Characteristics

**FIGURE 5:**
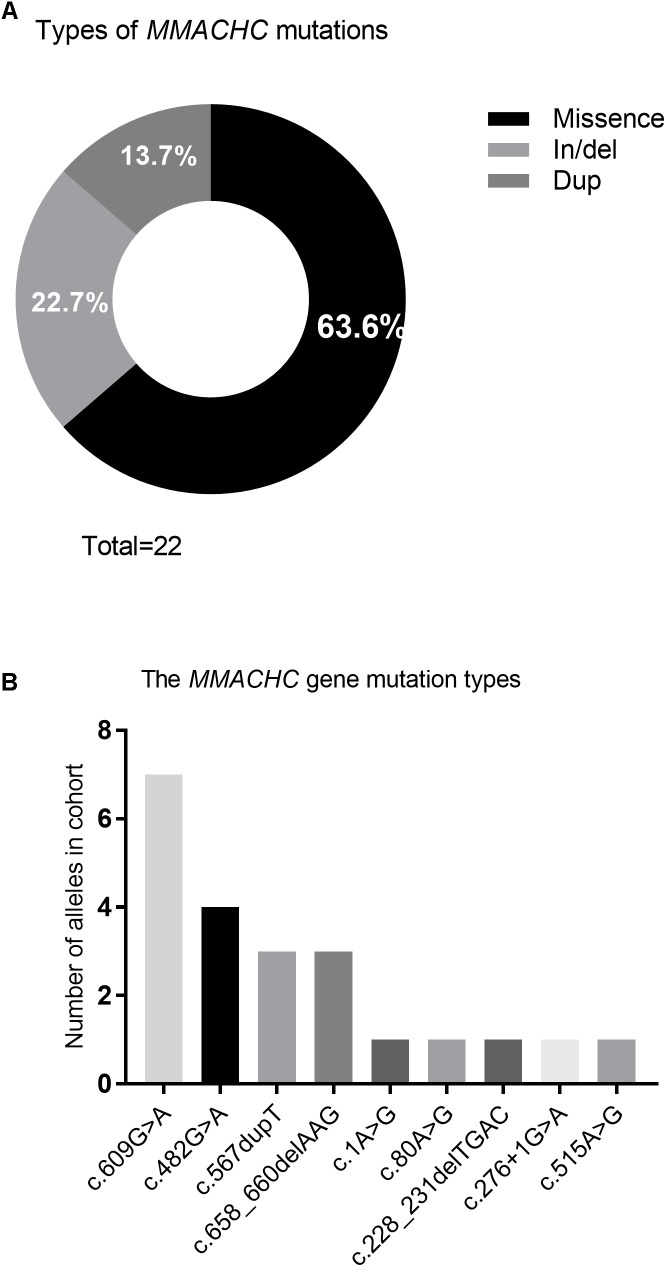
Frequency of variant types and mutations in *MMACHC* gene. **(A)** Pie chart summarizing the types of MMACHC mutations. **(B)** Frequency of alleles that identified in cohort.

**FIGURE 6:**
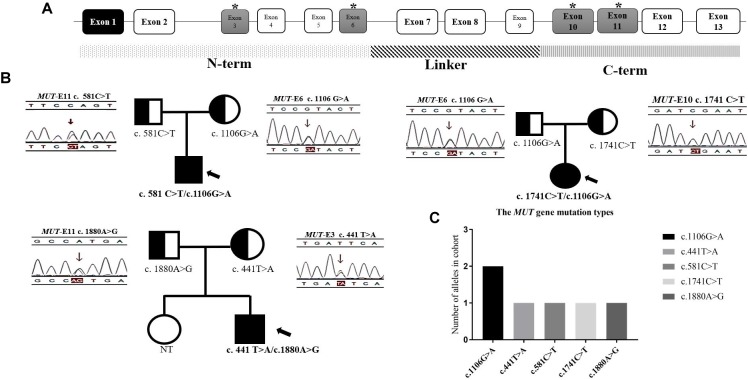
The distribution of mutations found in 3 *mut*-MMA patients. **(A)** The structure of *MUT* gene. **(B)** The family genetic map of three *mut*-MMA patients. **(C)** The frequency of *MUT* alleles. ^∗^ marked the exons/locations of the mutations identified upon the structure of *MUT* gene.

Nine different mutations in *MMACHC* (Figure 5 and Table 1) and five different mutations in *MUT* (Figure 6 and Table 3) were identified in 15 patients with MMA. Missense mutations were detected in the majority of cases and accounted for 63.6% of all *MMACHC* mutations (Figure 5A). Other mutations detected included insertions/deletions (∼22.7%), duplications (∼13.7%), splicing-related mutations and one initial codon mutation (Figure 5A). The c. 609G > A (p. W203^∗^) mutation was the most frequently identified mutation and was detected in 8 alleles (6 patients, 6 families) (Figure 5B and Table 1). The second most commonly detected disease allele was c.482G > A (p. R161Q) (4 alleles, 4 patients, and 4 families) (Figure 5B). Therefore, the two most frequent mutations found in our cohort were non-sense and missense mutations. A total of 11 clbC patients had two mutations and one individual had only one mutation in the *MMACHC* gene (Table 1).

Of the previously reported mutations in the *MUT* gene in our center, five different ones were identified in this study. All of the mutations were missense or non-sense mutations. The missense mutation c.1106G > A (p. R369H) was identified in more than one unrelated patient (Figure 6C). All of the mutations shown in Figure 6B were found in exons 3 and 6 or 10 and 11, which are located in the N-terminal and C-terminal domains, respectively (Figure 6A). In addition, the three isolated MMA patients were diagnosed with compound heterozygous changes in the *MUT* gene (Table 3).

## Discussion

Newborn screening by MS/MS revealed a small increase in the blood C3 concentration and an increased C3/C2 ratio. To explain the variations in C2, C3, and C3/C2, unaffected infants and MMA patients were compared during newborn screening. The usage of C2 significantly improved the screening sensitivity for MMA and reduced the number of false positives (Al-Dirbashi et al., 2016). The combination of abnormal C3 and C3/C2 results enabled the identification and referral of patients for further diagnosis (Armour et al., 2013). The presence of elevated MMA and homocysteine levels suggests an inborn error of cobalamin metabolism in clbC patients. In addition, MMA in the urine can result from both clbC disease and isolated MMA. According to our study, 236,368 newborns were screened, and 14 cases were diagnosed with MMA from November 2015 to December 2017 in Xuzhou. Thus, the incidence of MMA was approximately 1:16,883 in our area. The reported incidence of MMA ranges from 1:115,000 in Italy to 1:169,000 in Germany (Melo et al., 2011). However, there are no data available on the nationwide incidence of MMA in China. The estimated incidence of MMA was reported to be 1 in 26,000 infants in Beijing and Shanghai (Tu et al., 2013). In the Zhejiang Province, China, the incidence of MMA is 1:64,708 (Huang et al., 2011). In contrast, our study demonstrated an expectedly high incidence of MMA identified by MS/MS in Xuzhou.

Tandem mass spectrometry analysis of dried blood spots has become the predominant technique for newborn screening of inherited metabolic diseases. However, biomarkers in blood spots are susceptible to environmental conditions, such as heat and humidity. The most important and clinically relevant finding was that C2 levels declined sharply in the summer due to high air temperature and precipitations. This finding confirms with earlier observations that the storage of dried blood spots at room temperature in summer may accelerate C2 degradation, especially after blood collection in baby bathrooms. The stability of C2 and C3 is important for maintaining sample integrity for high-quality analysis and measurements of acylcarnitines in blood spots for MMA diagnosis (Adam et al., 2011). Ensuring optimal humidity and temperature conditions during dried blood spot transportation and storage is essential for maintaining sample integrity (Golbahar et al., 2014). To solve the above problems, the Cold-Chain Logistics system has been applied to improve dried blood spots storage conditions. Dried blood spot samples packaged with special sealed bags are stored in the 4°C constant temperature box, while transportation temperature is real-time monitored. Simultaneously, the effect of humidity and temperature can be avoided by the professional logistic method.

In the present study, we described the clinical, biochemical and molecular characteristics of a cohort of pediatric clbC patients treated at diagnosis. All of the patients were given hydroxocobalamin, folinic acid, levocarnitine and betaine following diagnosis. Of the 12 clbC patients who carried mutant alleles in *MMACHC*, two were homozygous and 9 were compound heterozygous for the mutations. Only one heterozygous change was detected in another patient, suggesting that the second mutation may be in a non-coding region. We found 9 different mutations of *MMACHC*, including c.609G > A, c.482G > A, c.567dupT, c.658_660delAAG, c.1A > G, c.80A > G, c.228_231delTGAC, c.276+1G > A, and c.515A > G (Liu et al., 2010; Wang et al., 2010). The most common mutation was c.609G > A, which reportedly has the highest incidence in Chinese clbC patients (Han et al., 2016). Four patients were heterozygous and two patients were homozygous for this mutation. The second most frequent mutation, c.482G > A, was detected in four patients (33.3%). In comparison, c.276+1G > A was reported as the most frequent mutation in European populations with clbC disease (Lerner-Ellis et al., 2009). Furthermore, we attempted to correlate the genotype with the phenotype in clbC disease. Presentation can occur during the neonatal period as an acute metabolic crisis or even death, as reported for case 3 in our center. The compound heterozygous mutations c.276+1G > A and c.658_660delAAG reportedly cause acute metabolic crisis at an early age. Moreover, insidious developmental delay without metabolic illness, or neurological deterioration, can also occur in a previously healthy child, adolescent or adult (Fischer et al., 2014). Seven patients who received treatment presented with various clinical manifestations, including intellectual disability or impairment (Kölker et al., 2015; Levenson, 2015). Case 2, who died a few months after birth, had the compound heterozygous mutation c.1A > G and c.658_660delAAG, suggesting that infant presentation should correlate with the presence of two mutations. Surprisingly, case 12, who was treated with only hydroxocobalamin due to the homozygous mutation c.609G > A, did not present with obvious developmental problems (Wang et al., 2010), whereas case 4, who had the compound heterozygous mutation c.609G > A and c.228_231delTGAC, had severe developmental delay, indicating that the c.228_231delTGAC mutation appears to be correlated with a more serious phenotype. What is not yet clear is the impact of these biochemical results on clbC individuals with only one mutation as Case 6. To date, there has been some reliable evidence that such compound heterozygotes might exit another mutation for a promoter epimutation, resulting from *PRDX1* mutations that force antisense transcription of MMACHC (Guéant et al., 2018). Although these represent the tip of the iceberg of potential reasons, indeed, it might be the possible explanation of the very interesting new syndrome of the patients with elevated tHcy. However, the number of patients in our study was limited, the information was not sufficient, and exact genotype-phenotype correlations cannot be established. Further investigations that examine environmental, ethnic and health condition including the retinal changes and individual genetic variations are warranted (Carrillo-Carrasco et al., 2012).

Three isolated MMA patients underwent *MUT* mutation analysis. Sequence analysis identified 5 different mutations of the diseased alleles, namely, c.441T > A, c.581C > T, c.1106G > A, c.1741C > T, and c.1880A > G, and all three patients had compound heterozygous mutations. The presence of CpG dinucleotides, namely, c.1106G > A and c.1741C > T in exons 6 and 10, respectively, was not reported to be population specific (Han L.S. et al., 2015). The other three mutations were located in exons 3 and 11. With respect to genotype-phenotype correlations, the limited number of patients included in our study, frequency of compound heterozygotes and lack of enzymatic detections made it difficult to assess the relationship between gene mutations and clinical manifestation. Patients P2 and P3, identified during the newborn screening with higher urinary MMA than P1, were both heterozygous for c.1106G > A. However, most patients homozygous for this mutation experience neonatal disease onset (Lempp et al., 2007). The *MUT* gene missense mutations in our study were all in the N-terminal and C-terminal domains (Forny et al., 2016; Han et al., 2017). This finding is consistent with the functional domain of MCM, and indicates that all the native amino acid residues involved are highly/fully conserved and likely to impact the structural stability of MCM (Acquaviva et al., 2005; Sakamoto et al., 2007; Zsengellér et al., 2014). Study limitations make an overall conclusion about MMA mutation types detection extremely difficult.

In conclusion, this is the first clinical cohort description focused on the inclusion of MMA as a target disorder for population-based newborn screening. MMA affects individuals worldwide but is an especially common inherited metabolic disease in our region. Based on our knowledge of MMA, patients should receive treatment as soon as possible after birth. No correlation was established between the associated mutations and clinical manifestations. Nevertheless, the molecular characteristics suggest the need for accurate and convenient genetic counseling for MMA patients and early prenatal diagnosis for high-risk families (Hu et al., 2018). Thus, it is critical to increase the coverage of newborn screening to allow early detection and treatment of MMA and combined MMA and homocystinuria patients.

## Ethics Statement

This study was carried out in accordance with the recommendations of ‘Expanding screening programs for neonatal diseases, Xuzhou Health and Family Planning Committee’ with written informed consent from all subjects. All subjects gave written informed consent in accordance with the Declaration of Helsinki. The protocol was approved by the ‘Xuzhou Health and Family Planning Committee’.

## Author Contributions

WZ and MG designed the experiments. WZ and HL carried out the experiments. WZ and CW analyzed the experimental results. XW worked as a clinical diagnosis and treatment doctor. HL assisted with collating clinical data. WZ and HL wrote the manuscript.

## Conflict of Interest Statement

The authors declare that the research was conducted in the absence of any commercial or financial relationships that could be construed as a potential conflict of interest.

## References

[B1] Acquaviva C. Benoist J. F. Pereira S. Callebaut I. Koskas T. Porquet D. (2005). Molecular basis of methylmalonyl-CoA mutase apoenzyme defect in 40 European patients affected by *mut0* and *mut-* forms of methylmalonic acidemia: identification of 29 novel mutations in the *MUT* gene. *Hum. Mutat.* 25 167–176. 10.1002/humu.20128 15643616

[B2] Adam B. W. Hall E. M. Sternberg M. Lim T. H. Flores S. R. O’Brien S. (2011). The stability of markers in dried-blood spots for recommended newborn screening disorders in the United States. *Clin. Biochem.* 44 1445–1450. 10.1016/j.clinbiochem.2011.09.010 21963384 PMC4557772

[B3] Al-Dirbashi O. Y. McIntosh N. Chakraborty P. (2016). Quantification of 2-methylcitric acid in dried blood spots improves newborn screening for propionic and methylmalonic acidemias. *J. Med. Screen.* 24 58–61. 10.1177/0969141316645824 27216769

[B4] Armour C. M. Brebner A. Watkins D. Geraghty M. T. Chan A. Rosenblatt D. S. (2013). A patient with an inborn error of vitamin B12 metabolism (cblF) detected by newborn screening. *Pediatrics* 132 e257–e261. 10.1542/peds.2013-0105 23776111

[B5] Carrillo-Carrasco N. Chandler R. J. Venditti C. P. (2012). Combined methylmalonic acidemia and homocystinuria, cblC type. I. Clinical presentations, diagnosis and management. *J. Inherit. Metab. Dis.* 35 91–102. 10.1007/s10545-011-9364-y 21748409 PMC4219318

[B6] Fischer S. Huemer M. Baumgartner M. Deodato F. Ballhausen D. Boneh A. (2014). Clinical presentation and outcome in a series of 88 patients with the cblC defect. *J. Inherit. Metab. Dis.* 37 831–840. 10.1007/s10545-014-9687-6 24599607

[B7] Forny P. Schnellmann A. S. Buerer C. Lutz S. Fowler B. Froese D. S. (2016). Molecular genetic characterization of 151 *Mut*-type methylmalonic aciduria patients and identification of 41 novel mutations in *MUT*. *Hum. Mutat.* 37 745–754. 10.1002/humu.23013 27167370

[B8] Golbahar J. Altayab D. Carreon E. (2014). Short-term stability of amino acids and acylcarnitines in the dried blood spots used to screen newborns for metabolic disorders. *J. Med. Screen.* 21 5–9. 10.1177/0969141314525367 24531510

[B9] Guéant J.-L. Chéry C. Oussalah A. Nadaf J. Coelho D. Josse T. (2018). A PRDX1 mutant allele causes a *MMACHC* secondary epimutation in cblC patients. *Nat. Commun.* 9:67. 10.1038/s41467-017-02306-5 29302025 PMC5754367

[B10] Han B. Cao Z. Tian L. Zou H. Yang L. Zhu W. (2016). Clinical presentation, gene analysis and outcomes in young patients with early-treated combined methylmalonic acidemia and homocysteinemia (cblC type) in Shandong province, China. *Brain Dev.* 38 491–497. 10.1016/j.braindev.2015.10.016 26563984

[B11] Han L. S. Huang Z. Han F. Wang Y. Gong Z. W. Gu X. F. (2017). Eight novel *MUT* loss-of-function missense mutations in Chinese patients with isolated methylmalonic academia. *World J. Pediatr.* 13 381–386. 10.1007/s12519-016-0085-z 28101778

[B12] Han L.S. Huang Z. Han F. Ye J. Qiu W.-J. Zhang H.-W. (2015). Clinical features and *MUT* gene mutation spectrum in Chinese patients with isolated methylmalonic acidemia: identification of ten novel allelic variants. *World J. Pediatr.* 11 358–365. 10.1007/s12519-015-0043-1 26454439

[B13] Han L. Wu S. Han F. Gu X. (2015). Insights into the molecular mechanisms of methylmalonic acidemia using microarray technology. *Int. J. Clin. Exp. Med.* 8 8866–8879. 26309541 PMC4538064

[B14] Hörster F. Baumgartner M. R. Viardot C. Suormala T. Burgard P. Fowler B. (2007). Long-term outcome in methylmalonic acidurias is influenced by the underlying defect (*mut0, mut*| [minus]|, *cblA, cblB*). *Pediatr. Res.* 62 225–230. 10.1203/PDR.0b013e3180a0325f 17597648

[B15] Hu S. Mei S. Liu N. Kong X. (2018). Molecular genetic characterization of *cblC* defects in 126 pedigrees and prenatal genetic diagnosis of pedigrees with combined methylmalonic aciduria and homocystinuria. *BMC Med. Genet.* 19:154. 10.1186/s12881-018-0666-x 30157807 PMC6116561

[B16] Huang X. W. Yang J. B. Tong F. Yang R. L. Mao H. Q. Zhou X. L. (2011). Screening for neonatal inborn errors of metabolism by electrospray ionization-tandem mass spectrometry and follow-up. *Zhonghua Er Ke Za Zhi* 49 765–770. 22321184

[B17] Huemer M. Scholl-Bürgi S. Hadaya K. Kern I. Beer R. Seppi K. (2014). Three new cases of late-onset *cblC* defect and review of the literature illustrating when to consider inborn errors of metabolism beyond infancy. *Orphanet J. Rare Dis.* 9:161. 10.1186/s13023-014-0161-1 25398587 PMC4255922

[B18] Kölker S. Garcia-Cazorla A. Valayannopoulos V. Lund A. Burlina A. Sykut-Cegielska J. (2015). The phenotypic spectrum of organic acidurias and urea cycle disorders. Part 1: the initial presentation. *J. Inherit. Metab. Dis.* 38 1041–1057. 10.1007/s10545-015-9839-3 25875215

[B19] Ktena Y. P. Paul S. M. Hauser N. S. Sloan J. L. Gropman A. Manoli I. (2015). Delineating the spectrum of impairments, disabilities, and rehabilitation needs in methylmalonic acidemia (MMA). *Am. J. Med. Genet. A* 167A, 2075–2084. 10.1002/ajmg.a.37127 25959030 PMC9017244

[B20] Lempp T. J. Suormala T. Siegenthaler R. Baumgartner E. R. Fowler B. Steinmann B. (2007). Mutation and biochemical analysis of 19 probands with *mut0* and 13 with *mut-* methylmalonic aciduria: identiWcation of seven novel mutations. *Mol. Genet. Metab.* 90 284–290. 10.1016/j.ymgme.2006.10.002 17113806

[B21] Lerner-Ellis J. P. Anastasio N. Liu J. Coelho D. Suormala T. Stucki M. (2009). Spectrum of mutations in *MMACHC*, allelic expression, and evidence for genotype-phenotype correlations. *Hum. Mutat.* 30 1072–1081. 10.1002/humu.21001 19370762

[B22] Levenson D. (2015). Medical foods prescribed to treat methylmalonic acidemia linked with adverse outcomes for some patients: studies explore impact of unbalanced amino acid formulation on growth, brain development. *Am. J. Med. Genet. A* 167A, ix–x. 26768187 10.1002/ajmg.a.37457

[B23] Liu M.-Y. Yang Y.-L. Chang Y.-C. Chiang S.-H. Lin S.-P. Han L.-S. (2010). Mutation spectrum of *MMACHC* in Chinese patients with combined methylmalonic aciduria and homocystinuria. *J. Hum. Genet.* 55 621–626. 10.1038/jhg.2010.81 20631720

[B24] Melo D. R. Kowaltowski A. J. Wajner M. Castilho R. F. (2011). Mitochondrial energy metabolism in neurodegeneration associated with methylmalonic acidemia. *J. Bioenerg. Biomembr.* 43 39–46. 10.1007/s10863-011-9330-2 21271280

[B25] Obeid R. Fedosov S. N. Nexo E. (2015). Cobalamin coenzyme forms are not likely to be superior to cyano- and hydroxyl-cobalamin in prevention or treatment of cobalamin deficiency. *Mol. Nutr. Food Res.* 59 1364–1372. 10.1002/mnfr.201500019 25820384 PMC4692085

[B26] Pastore A. Martinelli D. Piemonte F. Tozzi G. Boenzi S. Di Giovamberardino G. (2014). Glutathione metabolism in cobalamin deficiency type C (cblC). *J. Inherit. Metab. Dis.* 37 125–129. 10.1007/s10545-013-9605-3 23568438

[B27] Peng G. Shen P. Gandotra N. Le A. Fung E. Jelliffe-Pawlowski L. (2018). Combining newborn metabolic and DNA analysis for second-tier testing of methylmalonic acidemia. *Genet. Med.* 10.1038/s41436-018-0272-5 [Epub ahead of print]. 30209273 PMC6416784

[B28] Radmanesh A. Zaman T. Ghanaati H. Molaei S. Robertson R. L. Zamani A. A. (2008). Methylmalonic acidemia: brain imaging findings in 52 children and a review of the literature. *Pediatr. Radiol.* 38 1054–1061. 18636250 10.1007/s00247-008-0940-8

[B29] Richard E. Gallego-Villar L. Rivera-Barahona A. Oyarzabal A. Perez B. Rodriguez-Pombo P. (2018). Altered redox homeostasis in branched-chain amino acid disorders, organic acidurias, and homocystinuria. *Oxid. Med. Cell. Longev.* 2018:1246069. 10.1155/2018/1246069 29743968 PMC5884027

[B30] Sakamoto O. Ohura T. Matsubara Y. Takayanagi M. Tsuchiya S. (2007). Mutation and haplotype analyses of the *MUT* gene in Japanese patients with methylmalonic acidemia. *J. Hum. Genet.* 52 48–55. 10.1007/s10038-006-0077-2 17075691

[B31] Tu W.-J. Chen H. He J. (2013). Methylmalonic aciduria: newborn screening in mainland China? *J. Pediatr. Endocrinol. Metab.* 26 399–400. 10.1515/jpem-2012-0276 23446948

[B32] Wang F. Han L. Yang Y. Gu X. Ye J. Qiu W. (2010). Clinical, biochemical, and molecular analysis of combined methylmalonic acidemia and hyperhomocysteinemia (*cblC* type) in China. *J. Inherit. Metab. Dis.* 33(Suppl. 3), S435–S442. 10.1007/s10545-010-9217-0 20924684

[B33] Weisfeld-Adams J. D. Bender H. A. Miley-Akerstedt A. Frempong T. Schrager N. L. Patel K. (2013). Neurologic and neurodevelopmental phenotypes in young children with early-treated combined methylmalonic acidemia and homocystinuria, cobalamin C type. *Mol. Genet. Metab.* 110 241–247. 10.1016/j.ymgme.2013.07.018 23954310

[B34] Weisfeld-Adams J. D. Morrissey M. A. Kirmse B. M. Salveson B. R. Wasserstein M. P. McGuire P. J. (2010). Newborn screening and early biochemical follow-up in combined methylmalonic aciduria and homocystinuria, cblC type, and utility of methionine as a secondary screening analyte. *Mol. Genet. Metab.* 99 116–123. 10.1016/j.ymgme.2009.09.008 19836982 PMC2914534

[B35] Worgan L. C. Niles K. Tirone J. C. Hofmann A. Verner A. Sammak A. (2006). Spectrum of mutations in *mut* methylmalonic acidemia and identification of a common Hispanic mutation and haplotype. *Hum. Mutat.* 27 31–43. 10.1002/humu.20258 16281286

[B36] Yu H. C. Sloan J. L. Scharer G. Brebner A. Quintana A. M. Achilly N. P. (2013). An X-linked cobalamin disorder caused by mutations in transcriptional coregulator *HCFC1*. *Am. J. Hum. Genet.* 93 506–514. 10.1016/j.ajhg.2013.07.022 24011988 PMC3769968

[B37] Zsengellér Z. K. Aljinovic N. Teot L. A. Korson M. Rodig N. Sloan J. L. (2014). Methylmalonic acidemia: a megamitochondrial disorder affecting the kidney. *Pediatr. Nephrol.* 29 2139–2146. 10.1007/s00467-014-2847-y 24865477

